# Towards an equitable healthcare in China: evaluating the productive efficiency of community health centers in Jiangsu Province

**DOI:** 10.1186/s12939-017-0586-y

**Published:** 2017-05-25

**Authors:** Lulin Zhou, Xinglong Xu, Henry Asante Antwi, Linna Wang

**Affiliations:** School of Management, Jiangsu University, 301 Xuefu Road, Zhenjiang, Jiangsu People’s Republic of China

**Keywords:** Efficiency, Health resources, Urban communities, China’s Jiangsu Province

## Abstract

**Background:**

While the demand for the health service keeps escalating at the grass root or rural areas of China, a substantial portion of healthcare resources remains stagnant in the more developed cities and this has entrenched health inequity in many parts of China. At its conception, the Deepening Health Care Reform in 2012 China was intended to flush out these discrepancies and promote a more equitable and efficient distribution of health resources. Nearly half a decade of this reform, there are uncertainties as to whether the attainment of the objectives of the reform is in sight.

**Methods:**

We divided Jiangsu Province into 3 zones according to the level of economic and social development i.e. developed, developing, and undeveloped areas. Using a hybrid of Panel data analysis and an augmented Data Envelopment Analysis (DEA), we model human resources, capital inputs of Community Health Centers to comprehensively determine the technical and scale efficiency of community health resources in 3 zones in Jiangsu Province.

**Results:**

We sampled data and analysed efficiency and productivity growth of 75 Community Health Centers in 13 cities of Jiangsu Province from 2011 to 2015, which shows that a significant productive growth among Community Health Centers between 2011 and 2015. Mirroring the behavior of Community Health Centers, technological progress was the underlying force for the growth and the deterioration in efficiency change was found. This can be credited partly to the Deepening Health Care Reform measures aimed at improving technology availability in health centers in sub-urban areas. The regional summary of the DEA result shows that the stage of economic development and the efficiency performance of hospital did not necessarily go hand in hand among the 3 zones of Jiangsu.

**Conclusions:**

The government of China in general and Jiangsu province in particular could improve the efficiency of health resources allocation by improving the community health service system, rationalizing the allocation of health personnel, optimizing the allocation of material resources and enhancing the level of health of financial resources allocation.

## Background

The need to rejuvenate government effort and restructure public health functions to improve health equity in China is at the heart of healthcare reform in China [1]. This is captured in the preamble to the “Healthy China 2020” which is a national health reform policy program to provide universal healthcare access and treatment for all in China by the year 2020. Two years into the first major healthcare reform in China that started in 2009, China achieved universal health insurance coverage in 2011, representing the largest expansion of insurance coverage in human history [2].

Generally, China operates a three-tier or hierarchy of health care delivery system covering the urban and the rural areas [3]. The major primary healthcare providers in the rural areas are the village clinics and township health centers while county hospitals provide specialty medical services. In the urban areas however, the Community Health Centers (CHCs) and district hospitals provide primary healthcare while municipal and provincial hospitals offer tertiary medical services to both urban and rural people [4]. Eventhough the healthcare sector had been expanding since its inception, the 1970s, saw a more rapid growth in the number of institutions, workforce and challenges in the sector. For example at the end of 2015, there were nearly 1,006,000 health institutions, including 915,000 village clinics, 18,800 general hospitals and 54,300 CHCs [1]. The number of centers for disease control (CDCs) that provide public health programs to address infectious diseases, health education, food security, environmental health, etc were 54,600 while maternal and child care institutions and diseases specific treatment institutions were 30, 000 and 17,000 respectively. By 2015, the total number of health workers in village clinics had grown to about 1.87 million, with nearly 5.3 million others working in identified township and higher level health institutions [5]. Typical of most emerging and developing countries, a major challenge in the delivery of healthcare in China gross disparity in resources allocation between rural and urban areas. According to Li & Dong [6], most of the resources are concentrated in developed eastern provinces to the detriment of the poor western provinces. Even in developed province such as Jiangsu, there is a growing concern about equity in the allocation and efficient use of healthcare resources between rural and urban regions. Jiangsu is the fifth most populous and the most densely populated of all the provinces and autonomous regions of the People's Republic of China. It has the second highest GDP among Chinese provinces after Guangdong yet faced with many healthcare challenges. A 2015 report by the Provincial Department of Health showed that hospital bed utilization rate tumbled by nearly 0.98 percentage points in 2011 to 48.9% in 2013. This indicates a decline in the allocation efficiency of health resources [1] and this requires a more sustainable approach to ensure effective allocation of healthcare resources.

These and many other challenges facing the healthcare sector in Jiangsu Province significantly undermine the World Health Organization’s proposed health resources allocation principles of equity and efficiency [7]. In the past a number of systematic approaches were identified, proposed and applied to help improving efficient allocation of health resources under the deepening health care reform. Eventhough these approaches delivered different levels of successes, they were accompanied with myriad of implementation and sustainability challenges. Thus the hope that the Deepening Health Care Reform initiated in 2012 will bring efficiency in community health resources allocation has become a mirage owing to the conflicting views regarding the extent to which healthcare resources allocation has been improved under the new policy [8]. The search for alternatives has led many healthcare researchers to experiment with the Data Envelopment Analysis (DEA) model first proposed by Charnes et al. [9]. We employed a DEA model to analyse efficient utilization of human, financial and other resources among CHCs in Jiangsu Province because of its superiority over common or conventional efficiency measurement techniques (ratio and econometric regression methods). The DEA model is more consistent with economic theory because it locates technical or Pareto inefficiencies instead of measuring efficiency based on averages [10] (O'Neill, et al, 2008). DEA model does not cut-off points to be established to classify Decision Making Units (DMUs) efficiency levels [3] and permits multiple-input, multiple-output analysis. This can help managers to identify factors that exhibit high effect on operational efficiency. Moreover, since hospital input and output variables may be denominated in different units, the DEA model is preferred as it can accommodate input and output variables with different units of measurement. The DEA does not require a specific parametric functional form [10] (O'Neill, et al, 2008) and can be flexed to handle different economies of scale, conduct sensitivity analysis to determine areas with resources redundancies. Finally the DEA model was used in this study because it links all the factors of efficiency by evaluating the relationship between each input and output to arrive at scalar measure of performance efficiency [11].

However, the impact of policy variables can be shown through time series tests. This means that using DEA method alone without accounting for time variations may lead to spurious conclusions. To evaluate the impact of a policy initiative (Deepening Health Care Reform) that has been implemented over time, it is important to apply a functional form of DEA that allows for differences in efficiency measures over time to be accounted for. We therefore applied a hybrid of time series data and DEA model to evaluate resource allocation efficiency in the CHCs. This brings out the progressive, linear chart of broad time period of resource allocation and respective out turn. The time points efficiency variances measured and compared from year to year can reveal seasonal patterns that can serve as the basis for decision making. This type of information is of particular importance to the healthcare sector where seasonality induced healthcare hazards play a significant role in determining operational efficiency.

## Methods

### Data collection

**Fig. 1:**
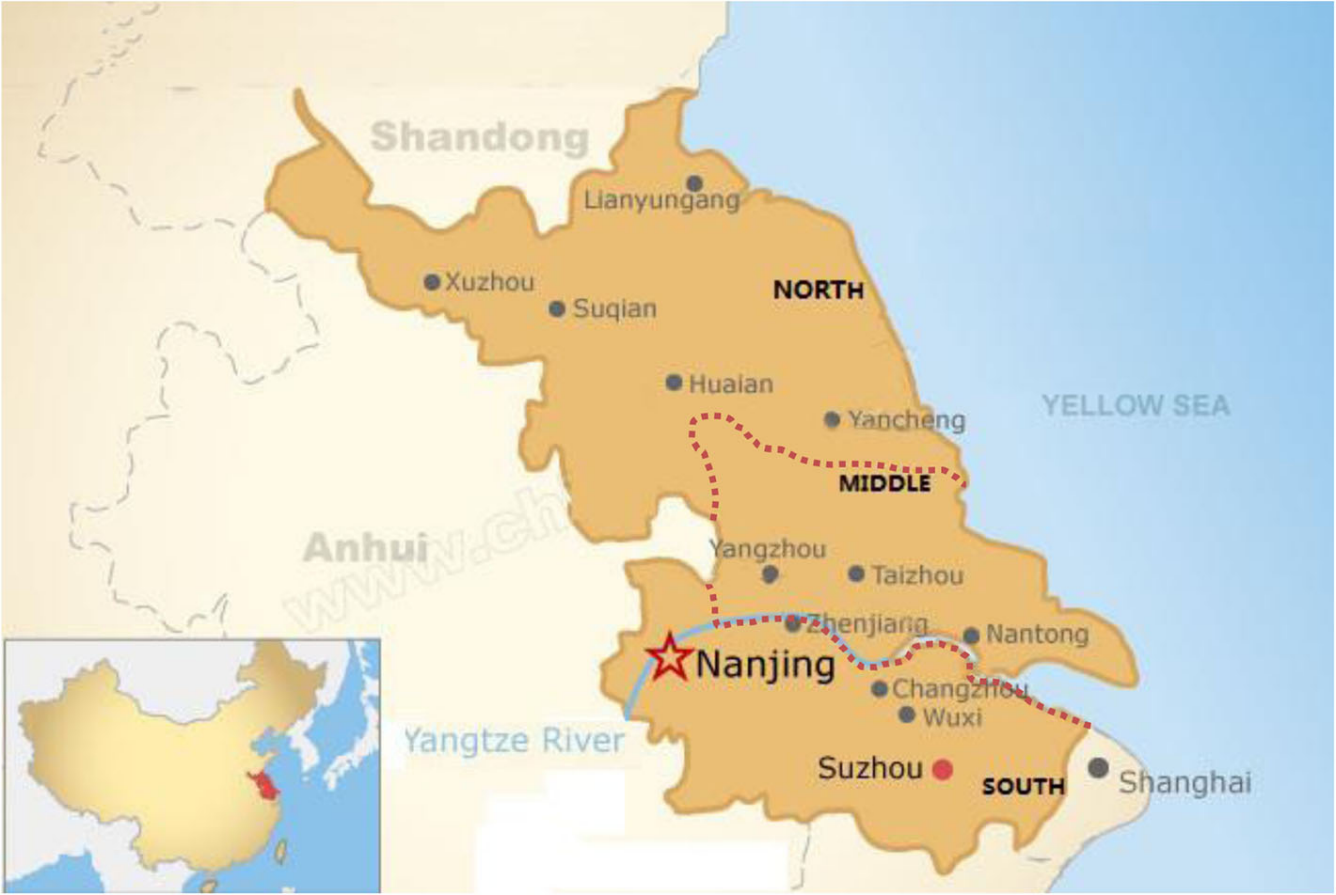
Geographical reference of data sources (Jiangsu Province)

There are many CHCs in Jiangsu Province but this study sampled data from 75 CHCs equally distributed in the three zones under review. Two reasons informed the selection of these specific 75 CHCs for the study. Firstly, they are designated as priority CHCs since 2013. For this reason, extended support in the form of technology, labour, capital, research and other valuable resources have been invested into them by the Provincial government to ensure high quality and more efficient services. Secondly, the 75 CHCs were chosen because they fall within the research jurisdiction of the Institute of Medical Insurance and Hospital Management of the Jiangsu University where this study was conducted. Research on other CHCs is conducted by other research centers in the province as part of measures to streamline data collection and research activities among designated organizations by the authorities. Twenty five (25) of the CHCs are in the most economically developed southern zone of Jiangsu Province (Nanjing, Zhenjiang, Suzhou, Wuxi, and Changzhou prefectures). Twenty five (25) of them are also located in Yangzhou, Taizhou, Nantong with relative economic development. These prefectures are located in the middle zone of the Jiangsu Province. Finally, the remaining 25 CHCs were sampled from Xuzhou, Lianyungang, Suqian, Huaian, Yancheng in the Northern zone of the Jiangsu province and are less economically active areas. The same number of CHCs was selected to provide a fair basis to make comparison of results. The Census and Statistics Department and the Jiangsu University which keeps validated and administrative data for these CHCs provided the input and output data. Also Zhang et al. [12] suggest that Jiangsu’s healthcare system is a microcosm of the pattern of healthcare delivery in China amidst its imbalance development across provinces. Thus identifying differences in Jiangsu and how that affects healthcare efficiency can give an idea of the performance of healthcare facilities in other provinces. Figure 1 shows the geographical mapping and reference of the parts of the Jiangsu Province from where data was collected for the study.

### Data selection

The study used an input and output form of DEA. Consistent with traditional production functions, labour and capital were treated as input variables in the production of health services in a Community Health Center. Labour was represented by (1) the number of doctors, (2) the number of nurses, (3) the number of pharmacists, and (4) the number of the other staff (medical staff and administrative workers). The number of beds was used as proxy for the CHCs capital stock (see [7, 13, 14]). On the other hand, the number of outpatient and inpatient cases was treated as outputs variables. We used DEAP 2.1 to conduct the regression of DEA model while Eviews 6.0 was used for the regression of the panel data model. To highlight the relationship between performance and economic development, the results were summarized by region in the next section.

### Data analysis

The input-output form of DEA model used in this research assumes that a Community Health Center (*j)* uses *N* inputs to produce M outputs. The input and the output vectors of a community health centre (j) is expressed mathematically as X_j_ = (x_1j_, x_2j_, …, x_Nj_) and Y_j_ = (y_1j_, y_2j_, …, y_Mj_) respectively. Thus the overall Farrell input-oriented technical efficiency measure (*TE*) can be computed using the following nonparametric frontier:

1 $$ \begin{array}{lll}\hfill & T{E}_j= min\leftthreetimes \hfill & \hfill \\ {}\mathrm{subject}\ \mathrm{to}\hfill & {\displaystyle {\sum}_{\mathrm{i}}{z}_i{y}_{mi\ge }{y}_{mj}}\hfill &\ \left( n=1,\dots M\right)\hfill \\ {}\hfill & {\displaystyle {\sum}_{\mathrm{I}}{\mathrm{z}}_{\mathrm{i}}{\mathrm{y}}_{\mathrm{mi}\le }{\leftthreetimes}_{\mathrm{j}}{\mathrm{y}}_{\mathrm{nj}}}\hfill & \left( n=1,\dots N\right)\hfill \\ {}\hfill & {\displaystyle {\sum}_{\mathrm{i}}{z}_i=1}\hfill & \left({z}_i>0\right)\hfill \end{array} $$

In these equations the efficiency estimates are based on the assumption that the relationships among the variables exhibits variable returns to scale (VRS). To compute efficiency estimates that assume constant returns to scale (CRS) the last constrained in expression (1) must be removed. The ratio CRS and VRS estimated efficiencies reflect scale efficiencies. This is expressed as:

$$ \mathrm{Scale}\ \mathrm{Efficiency}=\frac{\mathrm{CRS}\ \mathrm{technical}\ \mathrm{Efficiency}\ \mathrm{Score}}{\mathrm{VRS}\ \mathrm{technical}\ \mathrm{Efficiency}\ \mathrm{Score}} $$

We further decomposed the overall technical efficiency score into pure efficiency and scale efficiency as recommended by Baltagi et al. [3]. Pure technical efficiency denotes health decision making unit technical efficiency that cannot be attributed to deviations from optimal scale (scale efficiency) whereas scale efficiency measures the degree to which a health DMUs deviates from optimal scale. The optimal scale is defined as the region where the input-output relationship exhibits a constant return to scale). A non-parametric Malmquist index proposed by Färe, et al. [15, 16] was used to identify changes in productivity among the Community Health Centers (CHCs).

This index measure changes in the efficiency of a production unit transforming inputs into outputs from time *t* to time *t + 1*. Previous studies have expressed the Malmquist index in various distant function but the one adopted in this research is the most popular version which defines the input-based Malmquist index in period *t* as:

$$ {\mathrm{M}}_{\mathrm{o}}^{\mathrm{t}}=\frac{{\mathrm{D}}_{\mathrm{o}}^{\mathrm{t}}\left({U}^{t+1},{x}^{t+1}\right){x}^2}{{\mathrm{D}}_{\mathrm{o}}^{\mathrm{t}}\left({U}^t,{x}^t\right)} $$

Alternatively, for period t + 1,

$$ {\mathrm{M}}_{\mathrm{o}}^{t+1}=\frac{{\mathrm{D}}_{\mathrm{o}}^{t+1}\left({U}^{t+1},{x}^{t+1}\right){x}^2}{{\mathrm{D}}_{\mathrm{o}}^{t+1}\left({U}^t,{x}^t\right)} $$

where D_o_ represents the distant function and the superscripts represents the period of time that the efficiency values are being calculated. Specifically the superscripts on *u* and *x* represent the data period for the input and output data used to compute the efficiency scores. For D_o_^t + 1^(u^t^, x^t^), the Community Health Center data for period t + 1 is used. The final Malmquist index used is the geometric mean of the two indices expressed as:

2 $$ {M}_{\mathrm{o}}\left({U}^{t+1},{x}^{t+1},{U}^t,{x}^t\right)={\left[\left(\frac{{\mathrm{D}}_{\mathrm{o}}^t\left({U}^{t+1},{x}^{t+1}\right)}{{\mathrm{D}}_{\mathrm{o}}^{\mathrm{t}}\left({U}^t,{x}^t\right)}\right)\kern0.5em \left(\frac{{\mathrm{D}}_{\mathrm{o}}^{t+1}\left({U}^{t+1},{x}^{t+1}\right)}{{\mathrm{D}}_{\mathrm{o}}^{\mathrm{t}+1}\left({U}^t,{x}^t\right)}\right)\right]}^{\raisebox{1ex}{$1$}\!\left/ \!\raisebox{-1ex}{$2$}\right.} $$

This index avoids arbitrarily selecting one of the time periods as the reference point. This index can further be decomposed into two components to measure scale and technical efficiency differently as follows:

3 $$ {M}_{\mathrm{o}}\left({U}^{t+1},{x}^{t+1},{U}^t,{x}^t\right) = \left(\frac{{\mathrm{D}}_{\mathrm{o}}^{t+1}\left({U}^{t+1},{x}^{t+1}\right)}{{\mathrm{D}}_{\mathrm{o}}^{\mathrm{t}}\left({U}^t,{x}^t\right)}\right){\left[\left(\frac{{\mathrm{D}}_{\mathrm{o}}^{t+1}\left({U}^{t+1},{x}^{t+1}\right)}{{\mathrm{D}}_{\mathrm{o}}^{\mathrm{t}+1}\left({U}^{t+1},{x}^{t+1}\right)}\right)\left(\frac{{\mathrm{D}}_{\mathrm{o}}^t\left({U}^t,{x}^t\right)}{{\mathrm{D}}_{\mathrm{o}}^{\mathrm{t}+1}\left({U}^t,{x}^t\right)}\right)\right]}^{\raisebox{1ex}{$1$}\!\left/ \!\raisebox{-1ex}{$2$}\right.} $$

where $$ \left(\ \frac{{\mathrm{D}}_{\mathrm{o}}^{t+1}\left({U}^{t+1},{x}^{t+1}\right)}{{\mathrm{D}}_{\mathrm{o}}^{\mathrm{t}}\left({U}^t,{x}^t\right)}\right) $$ estimate the change in efficiency or the position of the production unit relative to the production frontier between time points *t* and *t + 1*. On the other hand $$ {\left[\left(\frac{{\mathrm{D}}_{\mathrm{o}}^{t+1}\left({U}^{t+1},{x}^{t+1}\right)}{{\mathrm{D}}_{\mathrm{o}}^{\mathrm{t}+1}\left({U}^{t+1},{x}^{t+1}\right)}\right)\left(\frac{{\mathrm{D}}_{\mathrm{o}}^t\left({U}^t,{x}^t\right)}{{\mathrm{D}}_{\mathrm{o}}^{\mathrm{t}+1}\left({U}^t,{x}^t\right)}\right)\right]}^{\raisebox{1ex}{$1$}\!\left/ \!\raisebox{-1ex}{$2$}\right.} $$ estimate the technical change (shifts in the production frontier between the time points). However this distance function expressed in Equation (2) also has a linear programming problem [15–19]. This can be ameliorated by decomposing the efficiency change into scale and technical efficiency change (Equations (4) and (5), respectively) as follows:

4 $$ \frac{{\mathrm{D}}_{\mathrm{ov}}^{t+1}\left({U}^{t+1},{x}^{t+1}\right)}{{\mathrm{D}}_{\mathrm{ov}}^{\mathrm{t}+1}\left({U}^t,{x}^t\right)} $$

5 $$ \Big[\frac{{\mathrm{D}}_{\mathrm{ov}}^{t+1}\left({U}^{t+1},{x}^{t+1}\right)/{\mathrm{D}}_{\mathrm{oc}}^{t+1}\left({U}^{t+1},{x}^{t+1}\right)}{{\mathrm{D}}_{\mathrm{ov}}^{\mathrm{t}+1}\left({U}^t,{x}^t\right)/{\mathrm{D}}_{\mathrm{oc}}^{t+1}\left({U}^t,{x}^t\right)}\  x\ \frac{{\mathrm{D}}_{\mathrm{ov}}^t\left({U}^{t+1},{x}^{t+1}\right)/{\mathrm{D}}_{\mathrm{oc}}^t\left({U}^{t+1},{x}^{t+1}\right)}{{\mathrm{D}}_{\mathrm{ov}}^{\mathrm{t}}\left({U}^t,{x}^t\right)/{\mathrm{D}}_{\mathrm{oc}}^t\left({U}^t,{x}^t\right)} $$

where subscripts *ov* and *oc* relate to the technologies that exhibit variable returns to scale and constant return to scale respectively. For ease of interpretation, the reciprocals of the computed indices are presented here. In other words, a value greater than 1 indicates productivity growth while a value less than 1 implies deterioration.

## Results

In Table 1 all the inputs allocated to the respective CHCs (irrespective of zone) grew continuously from 2011 and 2015. On average, the CHCs in the southern zone consumed more health inputs than others during the period while fewer inputs were consumed by CHCs in the northern zone. Incidentally the number of pharmacists in these northern CHCs declined drastically over time. Regarding the outputs, Table 2 shows that most of the CHCs in the three zones turned out more between 2011 and 2015 eventhough not proportional to the upward trend in the inputs received. Overall CHCs treated more patients across board except in 2012 and 2015 when outpatient numbers reduced in the mid-zone. The number of inpatients treated between 2013 and 2015 also reduced in the mid zone.

**Table 1:** Community Health Center input by region from 2011 to 2015

	Southern zone	Middle- zone	Northern zone
2011			
Number of doctors	108.64 (124.92)	91.46 (75.04)	65.12 (76.70)
Number of nurses	114.03 (142.98)	86.61 (99.34)	76.13 (101.97)
Number of pharmacists	23.82 (27.19)	26.03 (22.72)	18.11 (17.74)
Number of other staff	45.45 (51.28)	41.66 (40.49)	35.03 (42.77)
Number of beds	263.27 (293.35)	183.53 (177.37)	178.67 (227.29)
2012			
Number of doctors	113.99 (131.08)	96.61 (76.68)	65.38 (76.45)
Number of nurses	120.96 (150.49)	90.33 (101.42)	79.20 (106.10)
Number of pharmacists	24.09 (27.67)	27.00 (24.45)	17.17 (17.17)
Number of other staff	46.78 (54.40)	40.54 (36.80)	39.20 (49.17)
Number of beds	272.43 (298.99)	189.57 (185.16)	183.41 (231.38)
2013			
Number of doctors	138.92 (149.89)	96.86 (80.03)	67.40 (80.51)
Number of nurses	156.49 (194.47)	94.44 (103.13)	84.45 (122.39)
Number of pharmacists	28.14 (30.18)	26.49 (23.02)	18.32 (17.98)
Number of other staff	55.43 (64.54)	39.91 (33.78)	40.83 (54.16)
Number of beds	286.88 (319.39)	195.04 (185.91)	187.74 (243.37)
2014			
Number of doctors	160.35 (177.95)	98.89 (87.81)	69.87 (95.95)
Number of nurses	175.09 (210.01)	98.46 (110.44)	89.27 (129.17)
Number of pharmacists	28.96 (33.12)	24.45 (19.48)	16.29 (16.43)
Number of other staff	58.91 (64.23)	38.62 (36.99)	35.07 (47.88)
Number of beds	306.42 (336.77)	199.72 (188.48)	201.26 (260.82)
2015			
Number of doctors	156.78 (163.49)	100.44 (84.33)	71.13 (94.52)
Number of nurses	184.52 (216.58)	90.88 (107.79)	109.08 (219.54)
Number of pharmacists	30.12 (33.52)	24.30 (20.71)	16.30 (16.88)
Number of other staff	63.66 (74.82)	41.66 (41.29)	38.52 (59.24)
Number of beds	323.17 (352.76)	206.59 (195.04)	220.95 (284.52)

**Table 2:** Community Health Center output by region from 2011 to 2015

	Southern zone	Middle- zone	Northern zone
2011			
Number of outpatients treated	415443.00 (443102.45)	130649.35 (129884.62)	97829.24 (127678.84)
Number of inpatients treated	7399.56 (8436.20)	4779.33 (5655.05)	4183.06 (6745.93)
2012			
Number of outpatients treated	453061.69 (482991.17)	135897.25 (134747.30)	102745.80 (137877.10)
Number of inpatients treated	8048.00 (8958.04)	5030.10 (6144.57)	4454.95 (7173.06)
2013			
Number of outpatients treated	491763.43 (524629.77)	133940.04 (132661.98)	109064.17 (147501.68)
Number of inpatients treated	8680.52 (9601.73)	5606.72 (6875.79)	4873.99 (7849.25)
2014			
Number of outpatients treated	603252.83 (1049100.16)	148146.02 (140858.75)	116661.30 (161280.77)
Number of inpatients treated	9606.59 (10718.50)	8482.67 (16506.59)	5547.24 (9015.13)
2015			
Number of outpatients treated	676363.45 (1470044.55)	151725.69 (146355.18)	123562.72 (175891.95)
Number of inpatients treated	10587.92 (11929.22)	7242.60 (8831.23)	6502.30 (10169.34)

### Efficiency estimates

Table 3 shows the summary statistics or the geometric means for the overall efficiency of the CHCs. The information suggests that more inputs were consistently used in the CHCs in the southern zone and were relatively more efficient than others (overall efficiency).Similarly, the least efficient CHCs in the north consumed least amount of resources. The table also shows that the number of inefficient CHCs (efficiency scores less than or equal to 0.25). While the efficiency distribution in CHCs in the northern zone remained fairly stable between 2012 and 2013 that of the CHCs in mid-zone declined. The improvement levels however reversed across the CHCs in the three zones by 2014 eventhough the regional pattern had remained unchanged. This reversal was occurred because more CHCs attained very low efficiency scores (see Table 3). In summary, it can be said that after three years, the overall efficiency in CHCs has declined by 2014–15, particularly in the southern zone and the mid-zone.

**Table 3:** Measures of efficiency by region from 2011 to 2015

	Southern zone	Middle- zone	Northern zone
2011			
Overall efficiency	0.3549	0.2938	0.2026
Scale efficiency	0.6900	0.6354	0.4468
Pure technical efficiency	0.4991	0.4484	0.4400
2012			
Overall efficiency	0.3754	0.2710	0.1987
Scale efficiency	0.7162	0.6351	0.4352
Pure technical efficiency	0.5084	0.4140	0.4427
2013			
Overall efficiency	0.3897	0.3064	0.2223
Scale efficiency	0.7079	0.6579	0.4653
Pure technical efficiency	0.5340	0.4518	0.4636
2014			
Overall efficiency	0.2255	0.1762	0.1438
Scale efficiency	0.6847	0.5574	0.4063
Pure technical efficiency	0.3195	0.3066	0.3432
2015			
Overall efficiency	0.2259	0.1984	0.1587
Scale efficiency	0.6163	0.5772	0.3899
Pure technical efficiency	0.3555	0.3333	0.3948

**Fig. 2:**
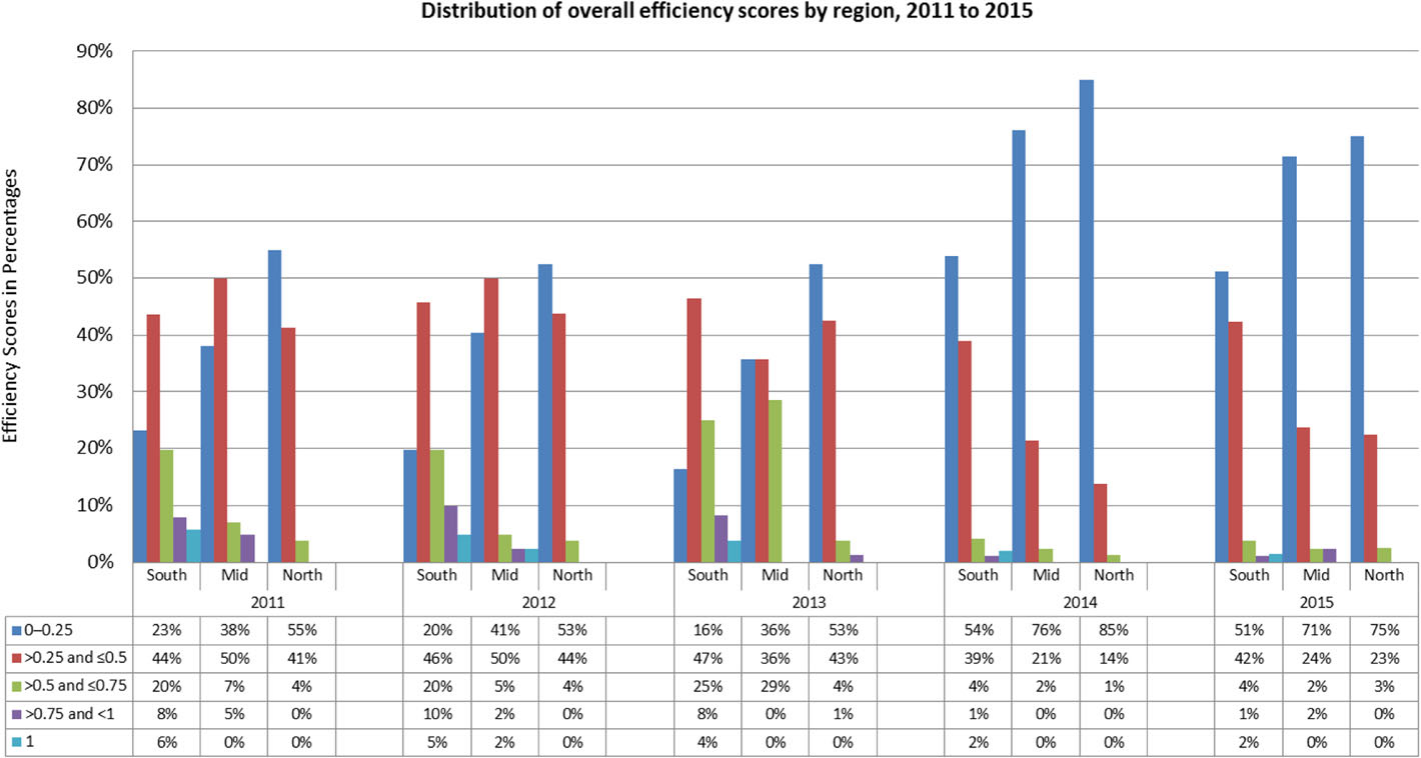
Distribution of overall efficiency scores by region from 2011 to 2015 (%)

**Fig. 3:**
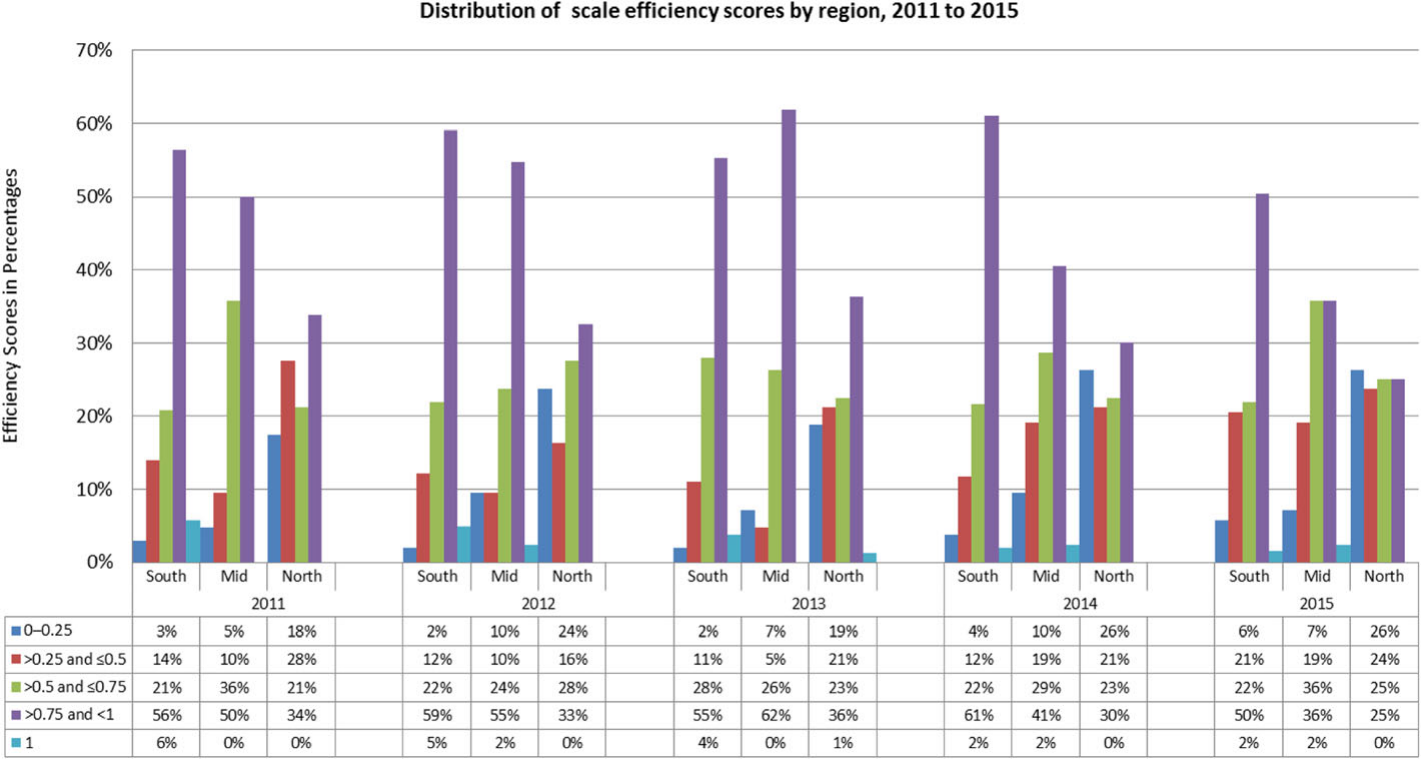
Distribution of scale efficiency scores by region, 2011 to 2015 (%)

**Fig. 4:**
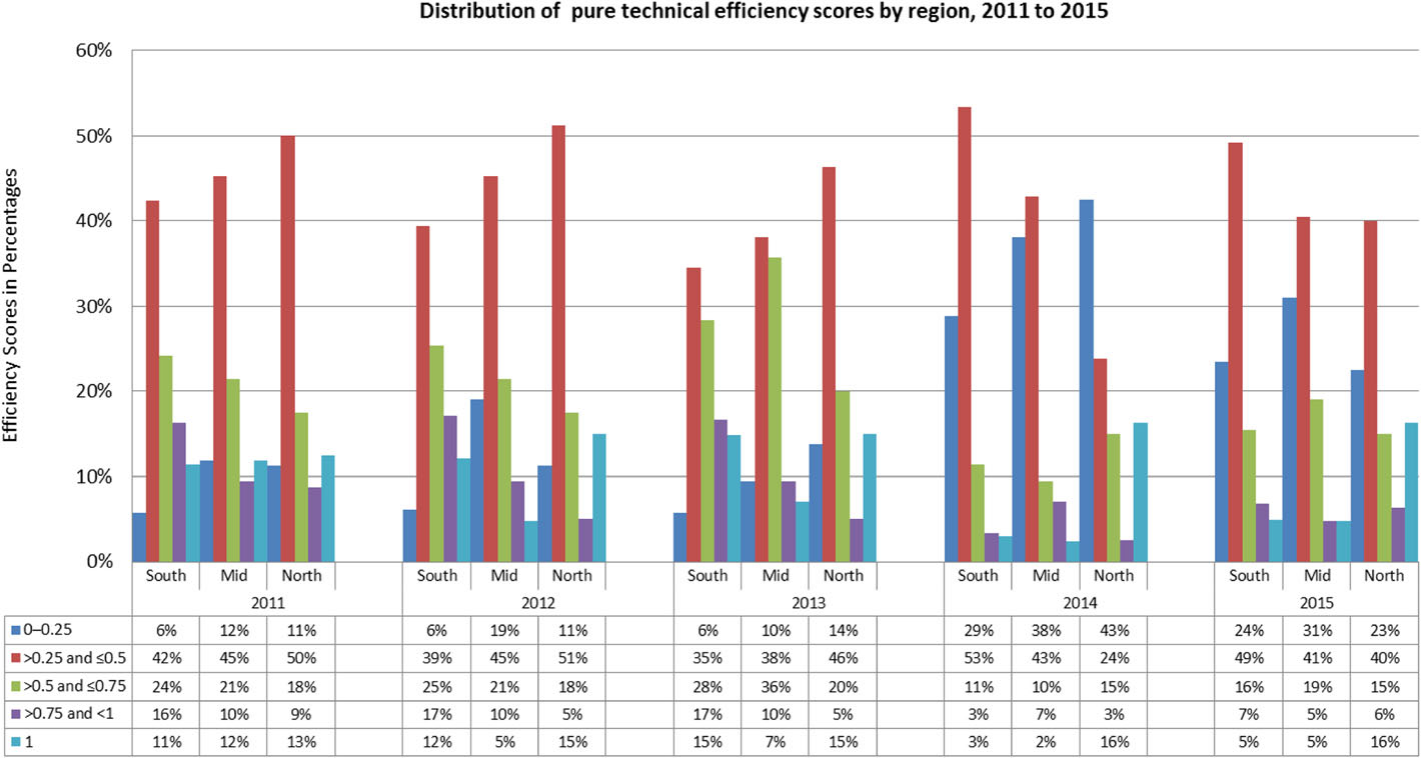
Distribution of pure technical efficiency scores by region, 2011 to 2015 (%)

The estimate of CHCs inefficiency in Fig. 2 indicates that the primary source of most CHCs inefficiencies in Jiangsu is pure technical inefficiency other than scale inefficiency except in the northern zone. The frequency distribution of pure technical and scale efficiency scores presented in Fig. 3 and 4 confirms this pattern already shown in Table 3. Fewer CHCs (especially in the mid-zone) attained pure technical efficiency score above 0.5 but CHCs in the Southern zones had a relatively better scale efficiency. For example, Fig. 3 show that more CHCs in the southern zones had high range efficiency values between 2011 and 2015. CHCs across the three zones experienced little scale efficiency before 2014. As shown in Table 3, scale inefficiency and pure technical inefficiency played more or less equal role in overall inefficiency for CHCs in the northern zone for the entire study period.

### Changes in productivity

Table 4 shows that on average CHCs productivity grew between 2011 and 2015 across the zones. Significantly, most of the CHCs in the rural areas in the northern zone experienced substantial growth in productivity. On average, productivity between 2011 and 2015 in the rural regions hovered around 31% to 39% relative to the much smaller productivity growth rate of 7% to 19% in urban areas over the same period. Eventhough Malmquist indices greater than 1 are recorded across CHCs in all the zones, a productivity growth pattern is noted in Table 4. For example while the year-to-year productivity change for CHCs in the southern zone show a downward trend, the overall productivity growth of CHCs in the northern zone was facilitated by the jump in productivity between 2013 and 2014.

**Table 4:** Malmquist productivity index and its decomposition by region, 2011 to 2015

	2011	2012	2013	2014	2015
Southern zone					
Malmquist index	1.0413	1.0407	0.9505	1.0254	0.9753
Technological change	1.6361	0.9842	0.9154	1.7720	0.9736
Change in efficiency	0.6174	1.0258	1.0072	0.5612	0.9717
Change in scale efficiency	0.8666	1.0069	0.9588	0.9382	0.8732
Change in pure technical efficiency	0.6911	0.9881	1.0190	0.5803	1.0795
Sample size	25	25	25	25	25
Middle-Zone malmquist index	1.1560	0.9762	0.9701	1.0835	1.0293
Technological CHANGE	1.7126	1.0583	0.8580	1.8847	0.9144
Change in efficiency	0.6548	0.8948	1.0968	0.5577	1.0919
Change in scale efficiency	0.8811	0.9693	1.0048	0.8218	1.0045
Change in pure technical efficiency	0.7209	0.8954	1.0588	0.6582	1.0543
Sample size	25	25	25	25	25
Northern-zone malmquist index	1.2793	1.0112	1.0194	1.1683	1.0042
Technological change	1.6337	1.0316	0.9107	1.8070	0.9098
Change in efficiency	0.7596	0.9508	1.0858	0.6271	1.0708
Change in scale efficiency	0.8465	0.9449	1.0369	0.8472	0.9307
Change in pure technical efficiency	0.8705	0.9760	1.0157	0.7181	1.1160
Sample size	25	25	25	25	25

Conversely, the 19% average growth rate of CHCs in the mid-zone was the good progress made in 2013–15. Moreover the decomposition of the Malmquist productivity index shows that the overall productivity growth of CHCs was facilitated by progress in technology over the period. Another observation shows that there was continuous improvement in change in year-to-year pure technical efficiency for CHCs in northern and western zones except 2013–14 during which all CHCs suffered a fall in change in pure technical efficiency.

## Discussion

### CHCs efficiency

Recalling previous works on efficiency estimates of CHCs or similar health facilities in other emerging economies, the average overall efficiency score of a healthcare facility of this nature in emerging countries ranges between 0.56 and 0.82 [20]. However our study returned a low efficiency score of 0.15 to 0.40 for CHCs in the Jiangsu Province. The study of Ng [21] regarding the efficiency of CHCs in Guangdong province may be a reliable nearest neighbor (provincial peer) to compare with the case in Jiangsu analysed in this research. The latter study returned a better overall efficiency for hospitals in Guangdong (15% to 40%). This indicates that the CHCs in the Jiangsu Province may be less efficient compared to those in other provinces in China. Indeed 21%–37% of the total inputs utilized by CHCs in Jiangsu in 2011 could have been enough to handle the same number of patient cases if the CHCs were more efficient. Yet, the inefficiencies were not all-period-round as symptoms of efficient improvement were recorded between 2011 and 2013 and positive productivity growth between 2011 and 2015 among the designated CHCs. In the absence of any information to the contrary, we can speculate that this is an indication that CHCs in Jiangsu benefited from the health care reform implemented in China over the previous two decades. This is consistent with the view of Kirigia, et al. [7] that reforms stimulate greater competitive environment and better functioning mechanisms that leads to enterprise efficiency. This notwithstanding, the unusual fall in the efficiency of the CHCs in Jiangsu by 2014, may reflect some weakness of the health care reforms implemented up to mid 2000s. This may explain the reason for a new set of provincial level reforms announced in 2009.

### CHCs productivity

The results about the growth in CHCs productivity can be compared to observed trends among similar healthcare facilities in Finland [22] ,Northern Ireland [23] and Austria [24, 25], Germany [13], Angola [7] and Ghana [14] albeit differences in the sources of growth in the respective countries. For example Pilyavsky, et al. [26] documented that despite the efficiency improvements in Ukrainian and South African healthcare facilities, they experienced technological retrogression. This is in sharp contrast to the case in Jiangsu province where in the midst of inefficiency there has been massive technological progress over the studied period. Indeed, 68% to 94% productivity growth between 2011 and 2015 was probably due to improved access to healthcare technology at the CHCs level as part of healthcare reform in China and this is reflected in the estimates of technological change in the Malmquist index. Secondly, the year-to-year fluctuations in scale efficiency may reflect the fluctuation in the demand for CHC services across different locations. The pattern of changes in technology and efficiency estimates indicate that technological improvement led to an out-shift of the production frontier. This means that the adoption of improved technology and improved therapy treatments and improved drugs at CHCs expanded or improved their feasible and attainable output. However, these medical inputs were inefficiently used to improve overall efficiency. The low overall efficiency estimates in Table 4 support this argument.

### Economic growth and CHC efficiency

Finally, economic imbalances across the different province in China are well documented in the extant literature. A significant number of studies on healthcare reform in China have identified a number of factors that negatively affects healthcare equity in the provinces. The inequity in relatively poorer provinces such as Tibet, Gansu, Xinjiang, Yunnan and Qinghai etc is largely because of limited resources and shortage of qualified health workers. More than 75% of the doctors working in village clinics are ‘barefoot doctors’ with very little medical training [21, 27, 28]. In these poorer provinces only 18.7% of township health workers are educated at medical university compared to 41% in cities [21, 29]. Meng [30] and Herd et al [31] observed that the demand for primary healthcare at the community level remains the largest source of healthcare demand but most of the resources are concentrated at the larger medical institutions in major cities and towns. This denies the largest proportion of those that need healthcare the ease of access. The analysis of the efficiency performance of the CHCs show variations based on development levels of the area. For example the more developed southern areas of Jiangsu recorded the best efficiency estimates. Similarly the CHCs in the middle zone also outperformed those in northern zone which is the most remote area.

## Conclusions

From the above exposition, we conclude that the government of Jiangsu province could improve efficiency of community health resources allocation in many ways. Firstly, there is the need to improve the community health service system. This can be done by strengthening the policy incentives to promote community care, promote cooperation between CHCs from different regions to actively share the medical resources. Further, it is suggested that health policy makers should ensure rational allocation of health personnel, strengthen community health service talent team construction. This includes setting up effective talent incentive mechanism, establishing a performance management system, improving the work efficiency of health personnel etc. This must be done in tandem with optimizing the allocation of capital and material resources such as technology and optimizing their configuration to suit the exigencies of the service profile of the CHCs. There is the need to enhance the level of health of financial resources allocation, explore the community medical financial investment, promote the basic system of community health service and, design efficient input-output evaluation mechanism to improve the operational efficiency of health resources fund.

## Abbreviations

CDCs: Centers for Disease Control
CHCs: Community Health Centers
DEA: Data Envelopment Analysis
DMUs: Decision Making Units
